# Supplementary figures and images for: Artificial Soils Reveal Individual Factor Controls on Microbial Processes
Source: mSystems. 2022 Jul 26;7(4):e00301-22. doi: 10.1128/msystems.00301-22 (PMC9426496; doi:10.1128/msystems.00301-22)

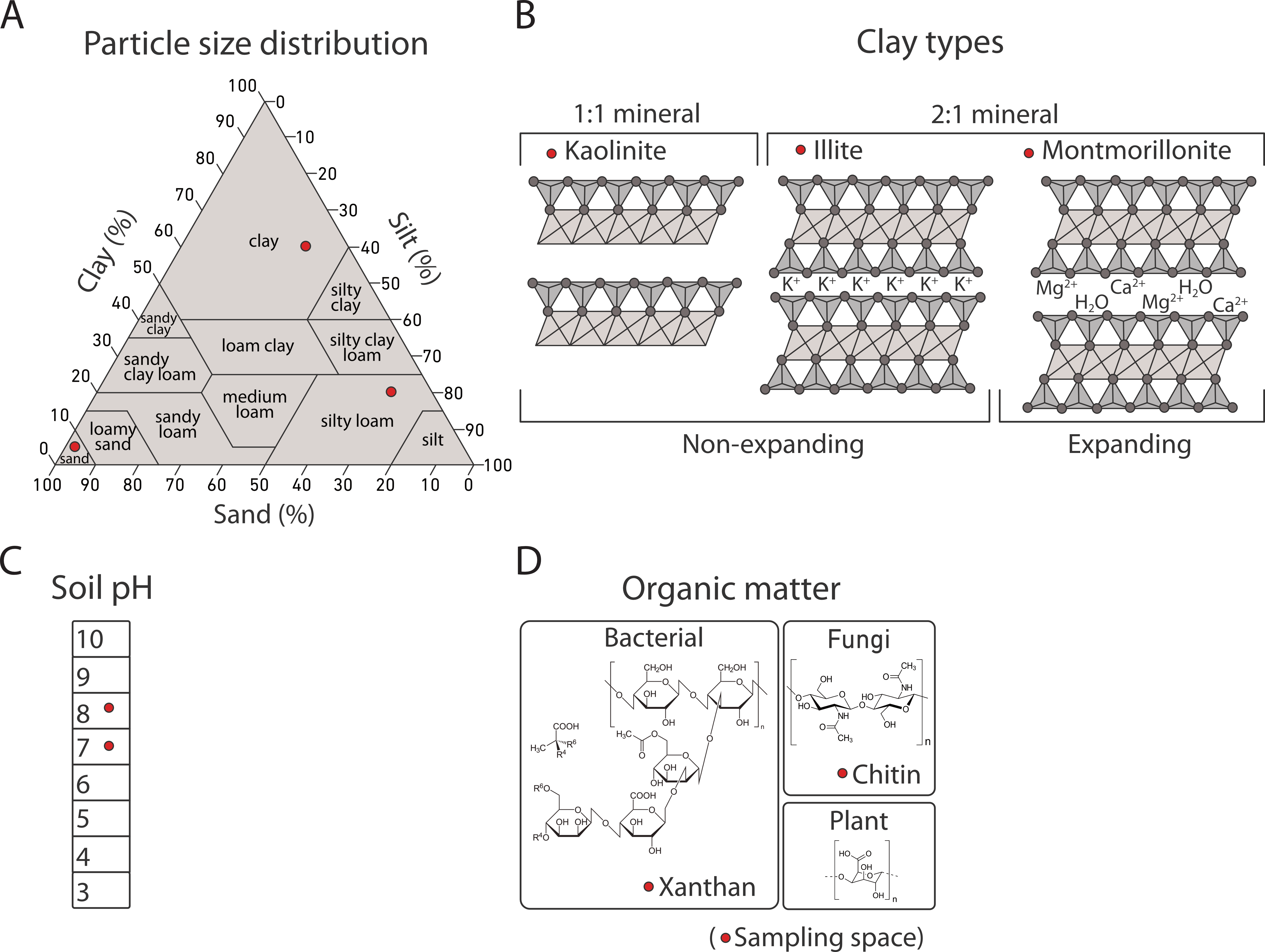

Supplement: FIG S1 [file msystems.00301-22-s0001.tif]

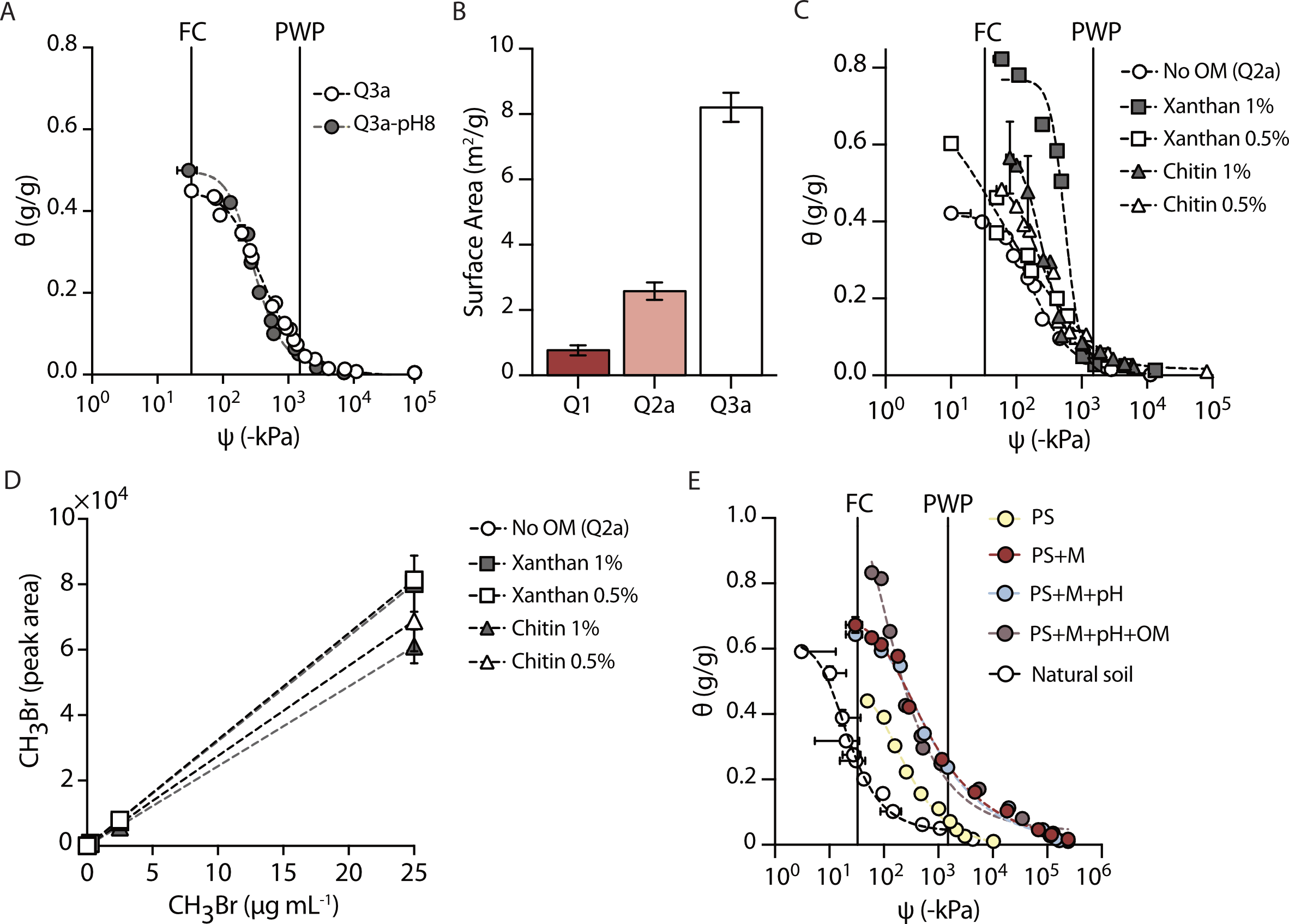

Supplement: FIG S2 [file msystems.00301-22-s0002.tif]

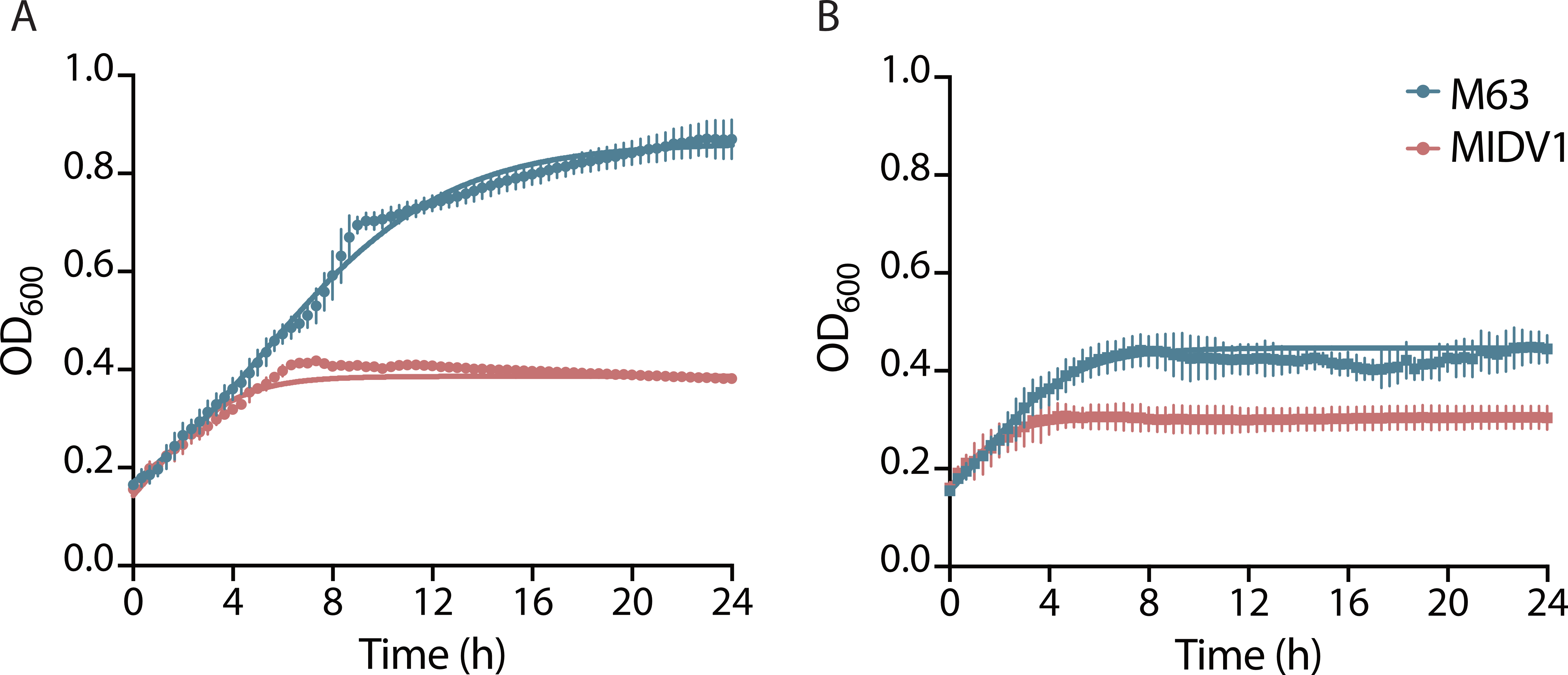

Supplement: FIG S3 [file msystems.00301-22-s0003.tif]

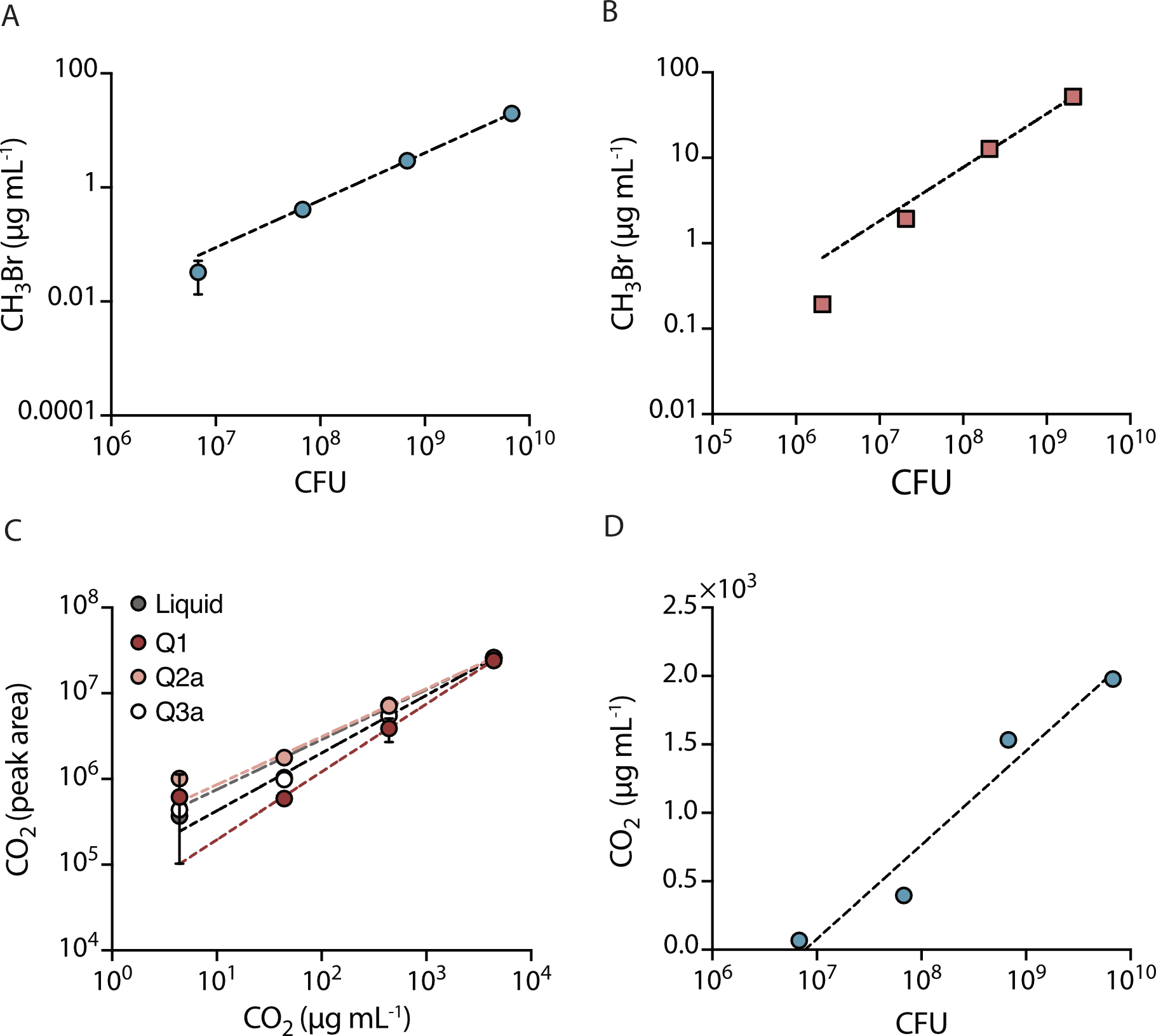

Supplement: FIG S4 [file msystems.00301-22-s0004.tif]

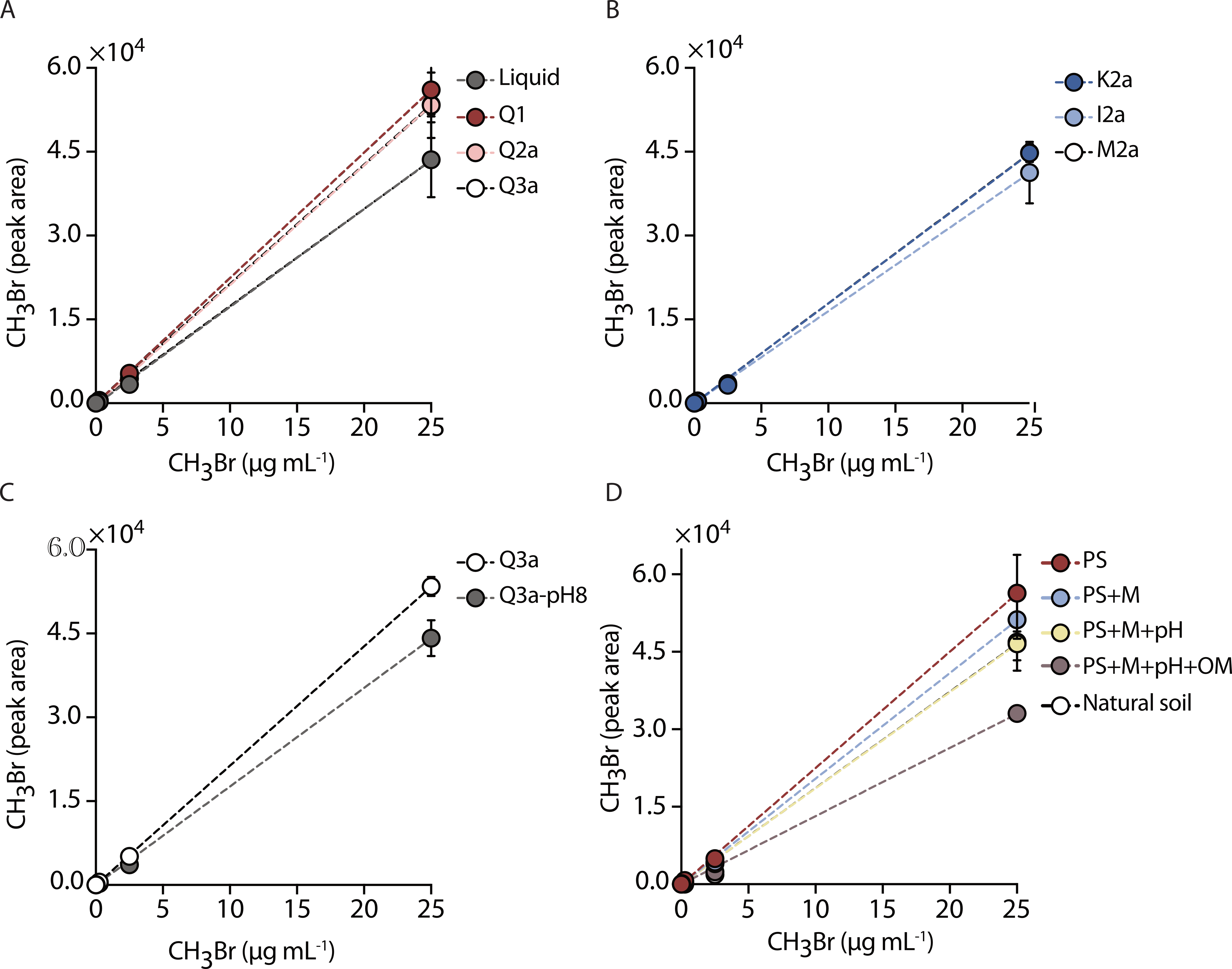

Supplement: FIG S5 [file msystems.00301-22-s0005.tif]

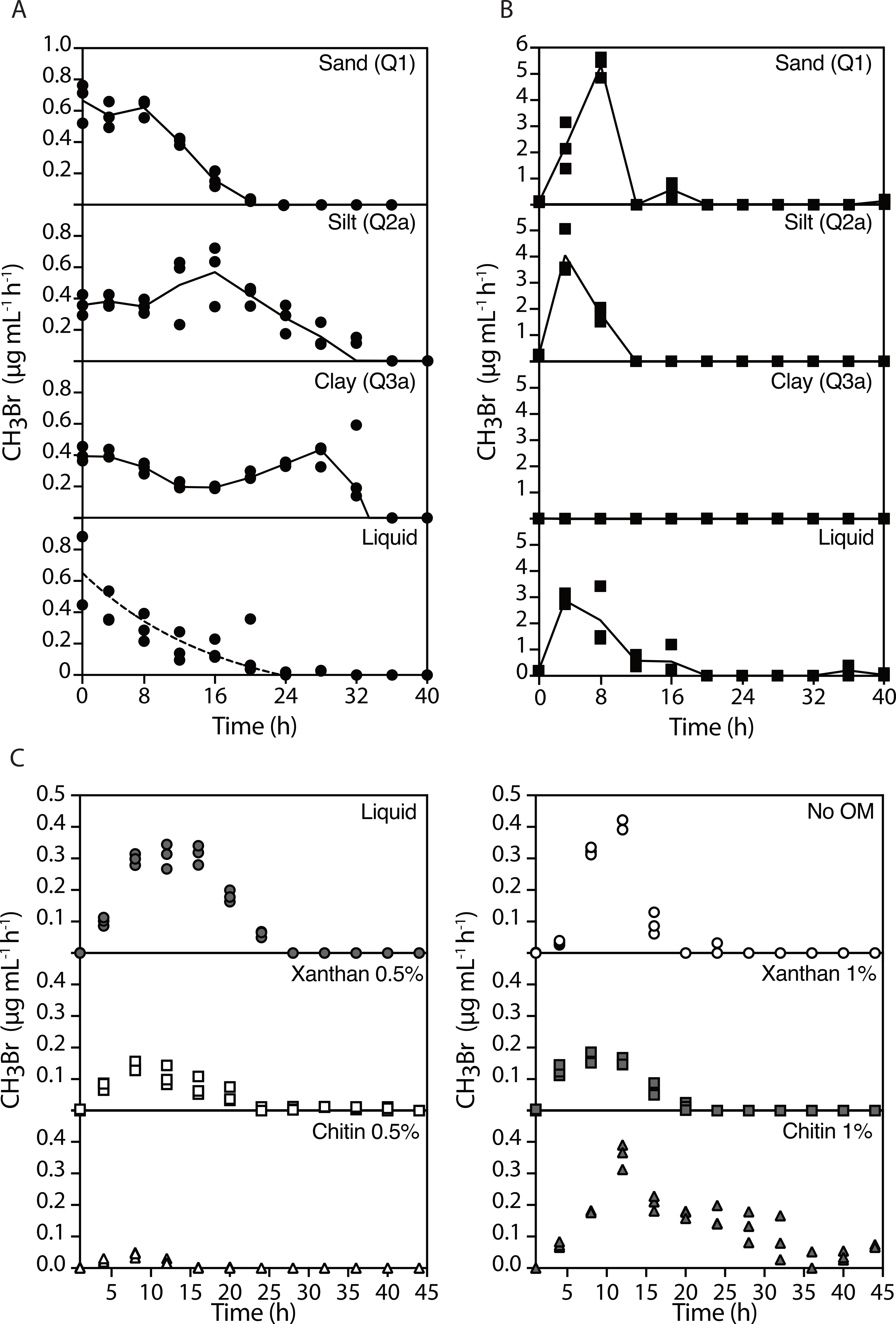

Supplement: FIG S6 [file msystems.00301-22-s0006.tif]

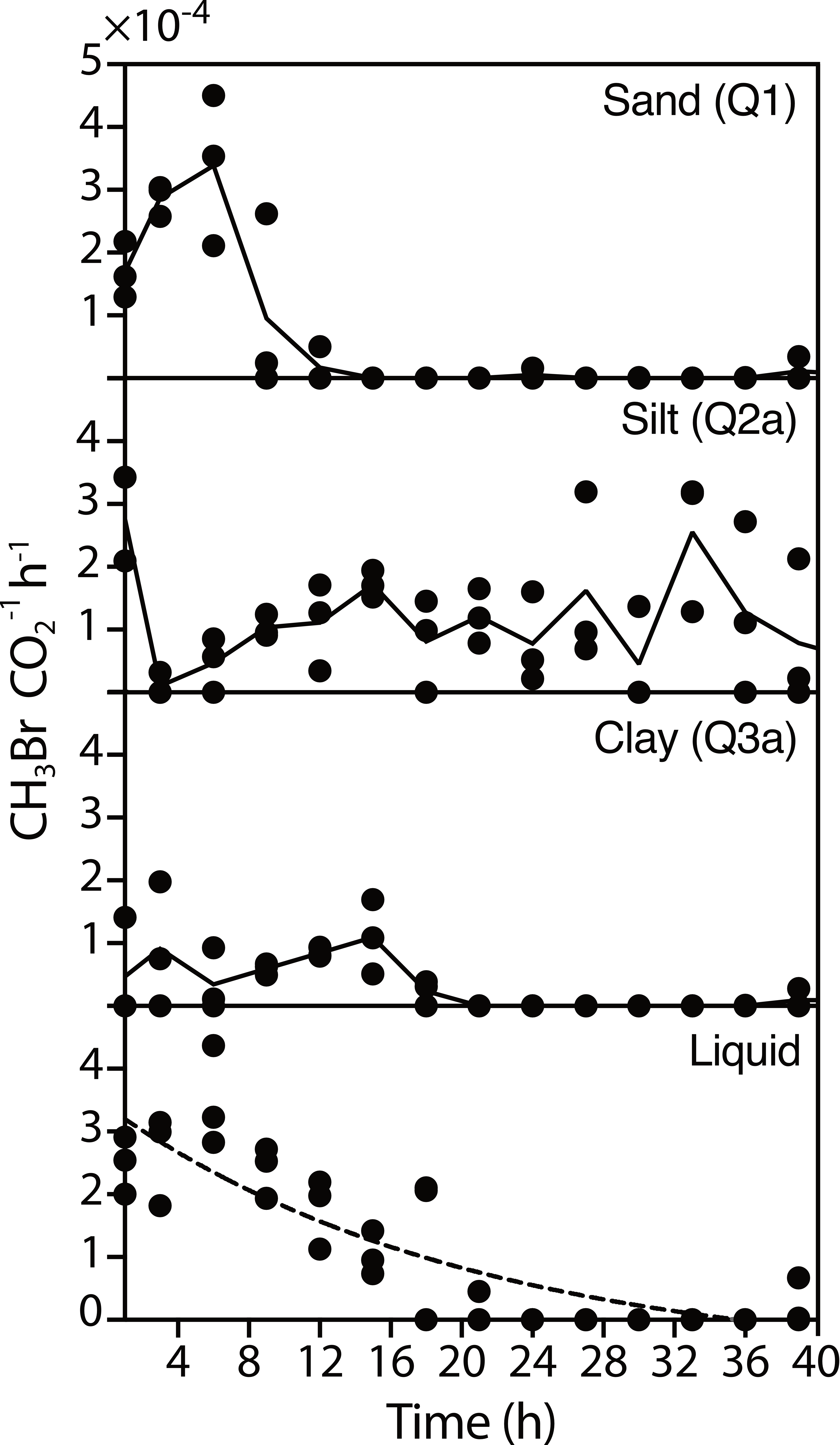

Supplement: FIG S7 [file msystems.00301-22-s0007.tif]

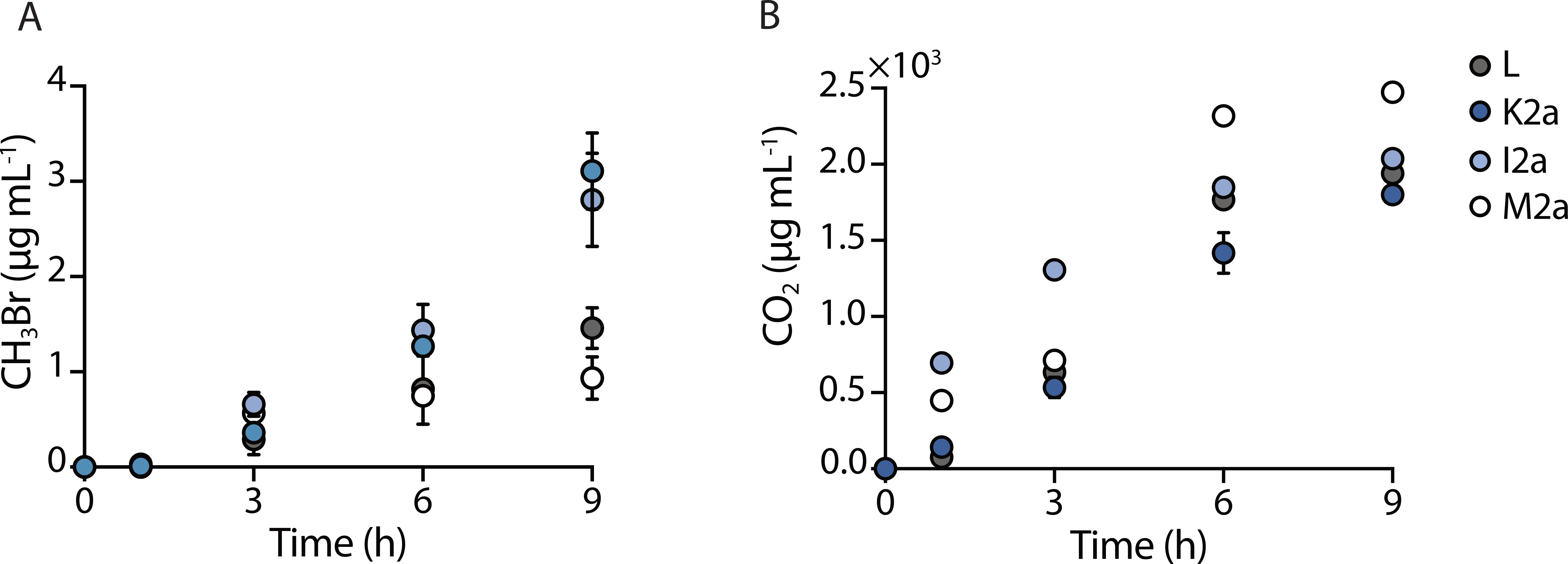

Supplement: FIG S8 [file msystems.00301-22-s0008.tif]
